# Developing implementer competence: trainees’ experiences during a training program for an emotion regulation intervention in schools

**DOI:** 10.3389/fpsyg.2026.1747220

**Published:** 2026-03-24

**Authors:** Lorena Wenger, Constanza Andrade Torres, Francisca Román Mella, Luis Painemal, Clementina Hueche, Rocío Briceño, Carla Rocha, Yuliana Ulloa, Sofía Villar

**Affiliations:** Departamento de Psicología, Universidad de La Frontera, Temuco, Chile

**Keywords:** adolescent mental health, emotion regulation, experiential learning, facilitator readiness, implementer training, reflexive thematic analysis, school-based intervention

## Abstract

Emotion regulation difficulties are central to adolescent mental health, and school-based interventions tackling these challenges rely on well-prepared implementers who can deliver skills confidently and sensitively within educational settings. However, little is known about how implementers experience their training or how this preparation influences their readiness to lead small, targeted groups in schools. This study explored the experiences of seven trainees who completed a two-phase preparation process for delivering a group-based emotion regulation program for adolescents. Using a qualitative design with reflexive thematic analysis, we examined individual interviews conducted after an experiential module, where trainees practiced the same skills they would later teach, and a group interview held after a pilot implementation phase focused on simulated delivery, group management, and feedback. Four themes emerged: critically assessing the training process; moving toward ownership of learning and becoming facilitators; valuing relationships as a foundation for learning; and anticipating challenges in the facilitator role, including student engagement, crisis moments, and school logistics. Overall, the findings demonstrate that implementer training is not solely technical but involves personal skill development, relational safety, and the ability to anticipate contextual demands. These insights can inform the design of training programs that strengthen implementer readiness and improve fidelity in school-based social and emotional interventions.

## 1 Introduction

Adolescence has been characterized as a period of heightened vulnerability for the development of internalizing problems and engagement in risk behaviors, in which difficulties in emotion regulation (ER) have emerged as a key transdiagnostic factor (McLaughlin et al., 2011; Aldao et al., 2016; Beauchaine and Cicchetti, 2019). ER is understood as the ability to initiate, maintain, and influence the expression of emotions, involving the use of various skills, including identifying affect in others, recognizing one’s own emotional state, and employing strategies to manage emotional responses (Houck et al., 2016; Bailen et al., 2019; McRae and Gross, 2020).

ER strategies are modifiable and can be developed through training in specific skills (Daniel et al., 2020; Pedrini et al., 2022; Holmqvist Larsson et al., 2023). Intervening in ER during adolescence constitutes a practical approach that has been shown to reduce symptoms of anxiety, depression, and risk behaviors among individuals who acquire new skills (Houck et al., 2016; Daniel et al., 2020; Eadeh et al., 2021; Pedrini et al., 2022).

Teaching ER skills is more effective when implementers not only understand their theoretical foundations but have also developed and applied these competencies in their own daily lives. Evidence suggests that implementers who enhance their emotional competencies are more effective in creating safe and effective learning environments and delivering social–emotional education programs with greater fidelity (Jennings and Greenberg, 2009; Brackett et al., 2011). Consistently, Linehan (2015) emphasizes that those who teach skills must actively practice them so they can transmit them with credibility, model their use in authentic contexts, and guide participants through direct experience.

This view aligns with a central principle of Implementation Science: implementer training functions as a competency driver. Within the Active Implementation Frameworks, competency drivers refer to structured mechanisms—such as selection, training, coaching, and performance assessment—that systematically develop practitioners’ capacity to deliver an intervention with fidelity (Fixsen et al., 2005; Metz and Bartley, 2012). In this sense, training is not merely informational but a foundational process for building the applied skills and performance capacities required for effective implementation. Thus, beyond the theoretical design of an intervention, the level and quality of implementation achieved directly influences outcomes (Durlak and DuPre, 2008), underscoring the importance of providing implementers with targeted training (Pedrini et al., 2022).

Although there remains little consensus on the best ways to train implementers—such as teachers or other educational agents—to apply evidence-based interventions in school settings with fidelity, flexibility, and sustainability (Crane et al., 2020), several key elements have been identified in the training process. For instance, Braun et al. (2024) suggest that implementers should be recognized as active participants in their own training process. Other studies highlight the importance of ongoing support and participation in supervision sessions with original experts during implementation (Pedrini et al., 2022; Evans et al., 2024). Likewise, training methods that combine interactive and multimodal learning techniques with supporting materials (e.g., brochures, manuals, or information folders) appear to be most effective (Pearce et al., 2012). In a study on implementers’ experiences in an emotional learning intervention, Mischenko et al. (2022) reported that practical activities—such as observing model sessions, role-playing, and receiving feedback—were particularly valued by participants as they prepared to deliver the intervention. Moreover, the quality of the relationship with trainers played a key role in the learning experience: trainers were perceived as more effective when they displayed a respectful, warm, and receptive attitude.

In sum, having a well-selected, supported, supervised, and adequately trained implementation team enhances outcomes for the ultimate beneficiaries (Nation et al., 2003). However, knowledge remains limited regarding how implementers experience and make sense of their training process, particularly in socio-emotional interventions. The literature on training has predominantly focused on quantitative outcomes—such as satisfaction levels, self-efficacy, or technical competence—while qualitative approaches exploring the learning experience and personal appropriation of required competencies are less common (Ritchie et al., 2020; Mischenko et al., 2022; Romney et al., 2022; Ojifinni et al., 2024). Understanding how future implementers experience their training allows for the identification of factors that facilitate or hinder their preparation, thereby contributing to the design of more effective training programs. This study examines the experiences of a group of implementers during their training process for delivering an emotion regulation intervention to adolescents.

## 2 Method

This study employed a descriptive qualitative approach aimed to explore the training experiences of future implementers (hereafter referred to as trainees) of an emotion regulation intervention for adolescents.

### 2.1 Context and description of the intervention

The training process was designed to prepare trainees to deliver a group-based emotion regulation intervention grounded in the Dialectical Behavior Therapy (DBT) model. The intervention was developed as an adaptation of the Dialectical Behavior Therapy Skills Training for Adolescents program (DBT-A; Rathus and Miller, 2015), tailored for a secondary prevention context in schools and targeting adolescents with subclinical symptoms of emotional dysregulation but without severe diagnoses. The adaptation was developed through consensus among the research team, prioritizing the most relevant skills for adolescents (Linehan, 2015) and optimizing its feasibility for implementation in educational settings.

The program consists of eight weekly sessions, each lasting 90 min, and is conducted in participating schools. The content is organized around the four core DBT-A domains: mindfulness (two sessions), emotion regulation (2), distress tolerance (1), and interpersonal effectiveness (2), plus a final session for integration and closure. Although DBT-A traditionally includes a fifth module, Walking the Middle Path, which focuses on parent–adolescent dialectics and family-based validation strategies, this component was not incorporated in the present intervention due to its school-based format and the absence of direct family involvement. Each session follows a standardized protocol defining specific objectives, practical exercises, homework review, supporting materials, and time allocations for each activity. Groups comprise approximately eight adolescents and are co-facilitated by pairs of trained psychologists. Within each pair, one facilitator leads the session while the co-facilitator monitors adherence to the protocol and assists in managing contingencies.

### 2.2 Training implementation

The first phase, Emotion Regulation Training, was an experiential training process designed to help trainees understand and practice the same ER skills they would later teach to adolescents. The goal was to promote internalization of ER strategies so that future instruction would be grounded in practical understanding. This phase was led by the principal investigator (Ph.D. in Psychology, who completed 65 h of Intensive Training in Dialectical Behavior Therapy through The Linehan Institute) and a co-facilitator with a Master’s degree in Clinical Psychology and over 15 years of clinical experience working with adolescents, as well as experience training clinical psychologists at the master’s level in the Chilean context. The process had three main objectives: (1) to strengthen trainees’ own emotion regulation capacities, (2) to foster an empathic understanding of the intervention by experiencing it from the perspective of its intended beneficiaries (the adolescents), and (3) to encourage the practical appropriation of the tools, thereby facilitating their later transmission with greater conviction, authenticity, and real-life examples.

During this phase, trainees reviewed all the skills included in the program following the same methodology they would use during the intervention. Each 2-h session began with a mindfulness practice, followed by the teaching of an ER skill—combining theoretical exposition with practical examples—and then a group practice activity. Each session concluded with an assignment encouraging participants to apply the new skill in daily contexts, along with a worksheet summarizing the key content covered. In subsequent sessions, following the initial mindfulness exercise, time was allocated for homework review to promote generalization of ER skills across contexts and to clarify doubts.

The second phase, Pilot Implementation, adopted a methodological and practice-oriented focus aimed at implementation readiness. Its main objective was to ensure that trainees acquired not only theoretical knowledge but also the pedagogical and group facilitation skills required to deliver the intervention sessions. To this end, trainees worked in pairs, simulating program sessions in which their peers acted as adolescents. These simulations included typical school-based challenges such as interruptions, questions, and contextual difficulties, allowing trainees to practice group management strategies, language adaptation, and handling of complex situations. Each session included feedback spaces focused on refining the intervention script, improving explanations of skills, and addressing technical questions. This process aimed to strengthen trainees’ implementation competencies while simultaneously contributing to the quality assurance of program materials and session guides.

### 2.3 Participants

A purposive sampling strategy was employed, including all individuals who participated in the training process to implement the group emotion regulation intervention for adolescents (trainees). The sample comprised seven participants—four final-year psychology students and three licensed psychologists—six women and one man, with a mean age of 22.5 years.

### 2.4 Data collection

**Figure 1:**
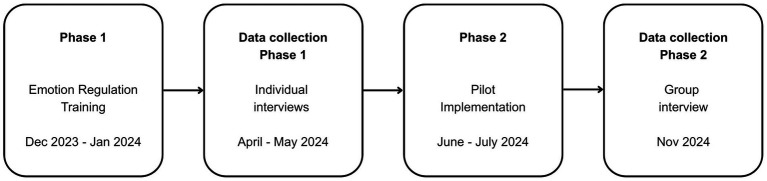
Timeline of the implementer training process and corresponding data collection points.

Data were collected using two techniques corresponding to each phase of the training process (see Figure 1). The interval between each training phase and data collection reflected the academic calendar and the sequencing of broader project activities. Each participant was contacted by the principal investigator, who explained the study’s objectives and invited them to participate. After completing the Emotion Regulation Training phase, individual interviews (II) were conducted following a semi-structured interview guide focused on trainees’ experiences during the training—such as key learnings or changes in attitudes and practices related to ER (e.g., “How would you describe your experience in the Emotion Regulation Training?” and “What challenges do you anticipate when implementing an emotion regulation intervention for adolescents?”). The interview guide was refined based on feedback from a psychologist with extensive clinical experience. Interviews lasted between 40 and 60 min.

Following the Pilot Implementation phase, a group interview (GI) was conducted with all participants to elicit their perspectives on specific topics in a more natural and interactive setting (Flick, 2007). The interview guide addressed aspects such as the overall evaluation of the training process, the main skills and/or competencies developed, anticipated implementation challenges, and areas for improvement in future training (e.g., “What skills do you think you developed during this second phase?”). The group interview lasted 94 min. The semi-structured interview guides for the individual interviews (Supplementary Table 1) and the group interview (Supplementary Table 2) are available in the Supplementary material.

Both individual and group interviews were conducted in person at the researchers’ university facilities. Before each interview, trainees provided written informed consent for participation. The interviews were conducted by one or two researchers—SV, RB, CR, or YU— who were not involved in delivering the training and had no supervisory relationship with participants. All interviews were audio-recorded and later transcribed for analysis. Interviews were conducted in Spanish. For publication purposes, participant quotations included in this manuscript were translated into English. Initial translations were assisted by generative AI (ChatGPT, version 4.1), and all translated excerpts were subsequently reviewed and refined by the research team to ensure semantic and contextual accuracy.

### 2.5 Ethical considerations

The study was approved by the Scientific Ethics Committee of the Universidad de La Frontera (Act No. 076_23, Record No. 038_23). Written informed consent was obtained for each data collection activity, specifying the study’s objectives, the voluntary nature of participation, the right to withdraw at any time, and the exclusive academic use of the data. Participant confidentiality was ensured through anonymization and secure storage of audio recordings.

### 2.6 Data analysis plan

While the analysis followed an inductive reflexive thematic approach, it was oriented around the following overarching research question: How do trainees experience and make sense of the process of preparing to implement an emotion regulation intervention in school settings? More specifically, the analysis explored: (1) which elements of the training process trainees identified as most significant; (2) what learning outcomes and competencies they perceived as having developed; (3) how prior personal or professional resources shaped their experience; and (4) what challenges they anticipated in implementing the intervention.

The preliminary transcription of the individual interviews and the focus group was conducted with the support of Google Pinpoint. All automatically generated transcripts were subsequently reviewed and manually corrected by members of the research team to ensure accuracy and fidelity to the original recordings.

Braun and Clarke’s (2021) approach to Thematic Analysis (TA) or Reflexive TA was used to analyze the data of this study. Reflexive TA is a flexible qualitative analytic method for developing, analyzing, and interpreting patterns of shared meaning (or themes) across a qualitative dataset. This involves following the six phases proposed by Braun and Clarke (2021), which guide a systematic and rigorous analytic process in which themes constitute the primary analytic outcome. In this approach, researcher subjectivity is an integral resource in the analytic process (Terry et al., 2017), and it was addressed through reflexivity exercises carried out during several team meetings throughout data collection and analysis.

The analytic flexibility of this methodological stance (Terry et al., 2017) enabled an inductive and organic approach to coding and theme generation. The analysis adopted a primarily semantic/descriptive orientation, with interpretive elements guided by the richness of the data. This theoretical flexibility also allowed for a contextualist epistemological stance, recognizing that access to reality is always mediated by the sociocultural meanings of both participants and researchers (Terry et al., 2017). Thus, while multiple representations or perspectives of reality may coexist, one version does not invalidate another (Madill et al., 2000).

Following the phases of this method, coding involved a systematic process in which each data item was examined and codes were assigned to all segments of the transcripts that conveyed potentially relevant meaning (Braun and Clarke, 2021). Each assigned code aimed to capture a specific and particular meaning. Consistent with Braun and Clarke’s conceptualization of coding as “an organic and evolving process” (2021, pp. 34), codes were iteratively refined as a deeper understanding of the dataset developed.

Coding was conducted collaboratively by RB, CR, YU, and SV to enhance comprehension, interpretation, and reflexivity (Braun and Clarke, 2021). Specifically, each interview was assigned to a pair of researchers who coded it independently and later compared their codes during synchronous meetings. The focus group was collaboratively coded after the initial theme generation, allowing the codes to complement and expand upon the preliminary themes.

In this study, a theme represented a shared pattern of meaning across the dataset, organized around a central concept (Braun et al., 2019; Braun and Clarke, 2021). Initial theme generation was conducted by the researchers, who coded the data by clustering codes with similar meanings. This process was supported and guided by LW, FM, CT, LP, and CH, who subsequently refined and deepened the preliminary themes through an exhaustive review of coded extracts and the full dataset to produce the final themes reported in this study. ATLAS.ti Web version 5.8.0 (ATLAS.ti Scientific Software Development GmbH, 2024) was used exclusively for data management and organisation; all analyses were conducted manually by the authors without AI-assisted features.

## 3 Results

### 3.1 Looking back: critically assessing the training process

This theme addresses the trainees’ evaluations of both phases of the training process—the Emotional Regulation Training and the Pilot Stage—considering aspects that facilitated their learning as well as those that could be improved in future training processes.

During the Emotional Regulation Training phase, trainees emphasized the theoretical and practical nature of the sessions, which not only allowed them to review the content but also to discuss and apply it themselves. As one participant explained, *“This workshop gave me information but also emphasized how I could deliver it in the future, and I think that is an important difference, because it’s not just about knowing—it’s also about learning how to do it”* (II). This format encouraged participation and sharing of experiences and perspectives: *“I really liked that it wasn’t just a class where I go, they give me something, and I leave—it was a space where I could participate and share my own experience”* (II).

Trainees also valued having assignments between sessions, as these kept them connected to the topic and allowed for ongoing reflection on the content. Reviewing the assignments at the start of each session helped them compare their work with that of others, as one participant described: *“That’s something I really highlight, because it allowed me to stay connected with what I was doing outside the classroom, and when coming back, not lose sight of where I was standing”* (II). The worksheets accompanying each skill were also viewed positively, serving as supportive materials and guides for completing the tasks.

Although the training process involved constant reflection on personal life aspects that could generate emotional distress, trainees appreciated the opportunity to share these experiences with others: *“Several times I thanked the guys for listening to me and for also talking about things that are hard to discuss… situations we all face every day that cannot really be changed, but we are learning tools to deal with them”* (II). Personal reflection, together with sharing experiences among trainees, led to greater knowledge and skill in ER, as another participant expressed*: “I think the skills I learned to regulate myself, to understand, to observe other people’s emotions better, to avoid judging, will help me—or could help me—not get frustrated in the teaching process”* (II).

Regarding the training structure, the first phase was intensive, with multiple sessions per week. This format made it difficult to complete assignments between sessions. Additionally, trainees felt that the session duration was sometimes insufficient to cover all planned content and review assignments thoroughly.

During the second phase—the Pilot Stage—focused on practicing session implementation, trainees noted that this process helped consolidate what they had learned earlier and allowed them to practice delivering content. One of the main challenges was taking on the role of an adolescent during simulations, as they needed to *“balance between not oversimplifying how an adolescent might think but also not assuming they know everything”* (GI). Although specific roles were initially assigned (e.g., *“the adolescent who does not pay much attention”* or *“the one who never brings the homework”*), the group later decided that representing adolescents more spontaneously was more effective and natural. Instead of adhering to predefined character types, trainees began responding in the moment as adolescents might—for example, asking for clarification when concepts felt too technical, requesting additional examples when something was unclear, or expressing confusion or mild resistance to simulate authentic classroom dynamics. They also recognized that this role challenged the skills of those acting as leader and co-leader: *“I think it was actually good because we kind of put the ones piloting to the test, like—‘what does that mean?’—asking questions that challenged some concepts, just to see how the facilitators reacted”* (GI).

Trainees highlighted that during pilot sessions, everyone could share opinions and provide feedback to the session leaders. They valued the respectful atmosphere in which feedback aimed to improve the sessions and scripts, helping fulfill the pilot’s purpose. As one participant said:

*Since I’ve used it and know it works, it felt important to convey that and ensure it reaches others. So, in that exercise—whether by watching my peers who had gone through the same process, or working together as a group—we also had the peace of mind that everyone was approaching it with a sense of responsibility, wanting to do it well, to improve, and to be constructive.* (GI)

Finally, trainees expressed concern that some sessions were not piloted or were carried out in a different order than planned. They emphasized that in future training processes, pilot sessions should follow the same order as the actual intervention, and more time should be devoted to reviewing mindfulness techniques and explaining the use of worksheets.

### 3.2 Moving toward ownership of learning: the process of becoming facilitators

This theme focuses on the meaningful learning experiences that trainees highlighted from their Emotional Regulation Training, particularly those they successfully integrated into their daily lives. It also includes learnings from the Pilot Stage, which allowed them to consolidate their skills through practical experience in simulated settings.

Trainees reported that the ER skills developed during the training had been useful both personally and professionally. They mentioned using techniques to manage their own emotions and applying them in their work with patients or groups, as one participant shared:

*I think it has helped me a lot professionally, especially during my internship. When I start feeling overwhelmed, I go back to this idea of mindfulness and focus on my breathing […] all these things that have helped me, I’ve shown to my patients, to the groups I work with—so I’ve really used them.* (II)

Among the most meaningful skills mentioned were mindfulness, wise mind—a DBT concept referring to the integration of emotional and rational states in decision-making—and DEAR MAN, an interpersonal effectiveness strategy used to assert needs and set boundaries effectively, due to their applicability in diverse situations and personal resonance: *“The ‘wise mind’ idea really clicked with me. It just made a lot of sense. I keep it with me—it kind of enlightened me, I’d say—it’s a concept I use a lot every day”* (II).

Trainees valued the opportunity to practice through role-playing during the pilot stage, as it helped them explore how to position themselves with adolescents and reflect on their attitudes. Although they acknowledged that simulations could not fully replace real work, they highlighted the importance of maintaining a respectful attitude toward adolescents and recognizing them as active agents: *“At some point before the pilot, we talked about it, like: Let us not underestimate the adolescents’ ability to understand what we are working on”* (GI).

During the Pilot Stage, these learnings were strengthened by facing more realistic situations, prompting a shift from personal skill development toward a more relational perspective. Trainees became increasingly aware that, since adolescents are the core and target of the intervention, the focus should be on understanding, anticipating, and responding to their needs, interests, and emotions.

From this standpoint, trainees strongly emphasized the need to design and implement interventions that are understandable, meaningful, and emotionally safe for adolescents: *“The key thing is this—being there for the adolescent, being present, making sure they are not alone”* (GI). They emphasized the importance of creating spaces where adolescents feel validated and heard, as this fosters trust. This was illustrated in the following reflection:

*So, if we make sure that what we’re doing makes sense or feels useful to them, I think they’ll pay attention and get engaged with what we’re working on. I’m not sure how it’ll play out in practice, but when I played the adolescent role, I felt that my doubts were heard, that my questions weren’t silly, and that if something wasn’t clear, that was totally fine—and I think, as an adolescent, that’s really important.* (GI)

### 3.3 The foundation of the training process: valuing relationships

This theme examines how trainees valued the relationships they formed among themselves and with their trainers, as well as the role these bonds played in their learning process—highlighting mutual support, peer validation, and consensus-building throughout the training.

The creation of a safe, trusting space where they could be vulnerable without judgment was described by one participant:

*Most people there were folks I hadn’t met before, but you could tell they were willing to support you if you needed it. It was a really welcoming space because we had to share very personal experiences with people we didn’t know, and being in that kind of environment—where nobody judged you, and they gave advice, shared their own experiences, and validated your feelings—was really nice.* (II)

Trainees described the training as a process characterized by respect and acceptance, both from trainers and peers: *“The training, in one way or another, ends up creating spaces for support, containment, and trust”* (II). They recognized that even though it can be hard to talk about personal experiences, everyone was willing to share and listen with empathy, as one trainee said: *“I think there was a lot of openness from everyone—being attentive, listening, empathizing with others’ stories or feelings, creating this atmosphere where people could share things”* (II).

Sharing personal experiences also facilitated learning and internalization of techniques, as one trainee noted: *“They used fun, everyday examples that kept you engaged and made you think about your own experience—like: Yeah, something similar happened to me once, and I reacted that way, but now that I know this, I’d act differently”* (II).

Trainees appreciated that the trainers promoted horizontal relationships within the group, fostering closeness and a collaborative environment: “I still call them ‘professors,’ but they’d say: No, guys, we’re a work team now. So, there wasn’t this hierarchy […] and because of that, we felt closer to them” (II). The empathy shown by the trainers and their willingness to share personal experiences contributed to an atmosphere of trust, which encouraged freedom to express opinions—even disagreements: *“That really helped because we could talk about different topics, or even say openly: You know what, I did not like that strategy or That did not work for me”* (II). They also felt confident that they could seek help if needed: *“I know that when I arrive at the session, if I’m struggling with something, I can communicate it, get support, and feel calm about it”* (II).

This sense of trust carried into the pilot stage, where it was key for collective decision-making. Trainees had to coordinate how to conduct simulations and adjust aspects of the intervention, reaching agreements on strategies, timing, and interaction styles. The openness to express and listen fostered respectful, horizontal dialogue. One trainee reflected: *“We worked really well as a team when making important decisions. Looking back, I think, wow—it was actually hard to get all of us to agree on one single decision”* (GI).

Preexisting bonds also facilitated honest and constructive feedback during the pilot stage. The ability to share observations without fear of judgment encouraged collaborative adjustments and strengthened group learning. As one trainee put it: *“Something I really value is how we gave feedback respectfully—because even when we disagreed, everyone stayed respectful, even when ideas were totally opposite”* (GI).

Finally, trainees recognized the importance of these interpersonal bonds and anticipated the need to promote similar climates when working with adolescents. They emphasized creating spaces of respect and care that encourage active participation and trusting relationships, as reflected in the following comment:

[During role-playing] *In moments that were a bit tense or challenging, it was great… like, if someone was talking too much, they’d be called out, but in a way that didn’t make them feel bad. It was always done with respect, based on the bond that had been built. That was great to experience, because if we’d handled it more from an authoritarian approach, the atmosphere in the sessions would’ve been totally different.* (GI)

### 3.4 Looking ahead: anticipating challenges in the facilitator role

Throughout the training, trainees began anticipating potential challenges they might face when implementing the intervention with adolescents in school settings. Based on their experiences across both phases, they identified potential personal and operational challenges, as well as the need for targeted strategies to address them. This theme summarizes their main concerns, along with the resources, learnings, and tools they considered key for facing future implementation with greater confidence and flexibility.

One anticipated challenge related to characteristics of adolescence itself, such as lack of motivation, distrust, or disinterest. These attitudes were seen as possible obstacles to active participation in sessions: *“Maybe they’ll find the activities or theory boring, maybe it will not make sense to them, or they’ll leave with a lot of questions”* (II). Although trainees initially felt confident that these difficulties could be managed through the ER skills they had acquired and by building rapport with adolescents, the pilot stage showed a need for more specific training on strategies to engage and sustain attention among adolescents with fluctuating motivation levels. As one participant said: *“I’ve been missing that other side—students who respond like: ‘I do not know,’ or ‘I do not care’… how to motivate them or show them that this can actually be useful”* (GI).

They also identified potential operational obstacles, including irregular attendance, inadequate physical spaces, and limited session time. These anticipated difficulties made them value flexibility and improvisation as key competencies for adapting the plan to emerging needs while maintaining program goals. As one trainee expressed: *“Improvising—and understanding that improvising does not mean going against what you planned—is a skill that I think would be super useful”* (GI). In this context, coordinated work between leader and co-leader was perceived as a crucial support resource: *“Luckily, we’ll have a leader and a co-leader—so we will not be alone running the class, and we can check in with each other”* (GI).

Across both phases of the training, concerns emerged about how to handle critical situations, such as disclosure of abuse or suicidal ideation. Although trainees had some knowledge of how to respond, they emphasized the need for explicit and accessible guidelines for the facilitator team. As one participant noted: *“Disclosures of abuse, maltreatment, suicidal thoughts… all those things need to be handled with the importance they deserve […] it’s important to have clear steps to follow when those things happen”* (GI). Accordingly, they suggested that future training should include specific crisis management instruction, as well as strategies for dealing with individual or group emotional dysregulation.

Despite these anticipated difficulties, trainees also identified elements that reinforced their confidence for future implementation. The pilot experience—especially the role-playing—helped them develop skills such as public speaking, problem-solving, and language adaptation, which reduced their initial anxiety about facilitating sessions. As one participant shared:

*At the beginning, just thinking about it made me anxious—like, ‘oh my god, I’m nervous, excited but scared’—and now it’s more like, ‘yeah, cool!’ I still feel excited, but not that anxious, fearful activation anymore, like I’m going to mess something up.* (GI)

Finally, they valued their newfound ability to stay present during implementation—attentive to what happens in the session without being dragged by anticipatory anxiety or fear of what might go wrong. This ability, closely tied to mindfulness and distress tolerance practices covered in ER training, enabled them to recognize their own emotional reactions and anticipate more conscious and effective responses. As one participant explained: *“Seeing what affects you, how it affects you, and how to work through it—being aware of your emotional reactions gives you the ability to keep them in mind and act from there”* (GI).

Thus, the training not only strengthened their technical competencies but also fostered openness and flexibility—key attitudes for intervening confidently even in uncertain contexts.

## 4 Discussion

This study provides evidence on the training process of emotional regulation (ER) implementers, showing that preparation extends beyond the technical mastery of the intervention and emerges as a process of personal appropriation, relational transformation, and anticipation of contextual barriers. Overall, findings show that training implementers involves not only the acquisition of technical knowledge but also personal, relational, and anticipatory processes that shape their readiness for real-world delivery.

According to the trainees, the integration of theory and practice was a key element for understanding and exercising ER skills. This aligns with Fixsen et al. (2005), who concluded that effective training processes involve presenting information (knowledge), providing demonstrations, and ensuring opportunities to practice relevant skills within the training environment (behavioral rehearsal). This finding is consistent with the trainees’ descriptions of the Piloting phase, which allowed them to apply what they had learned and simulate real-life intervention situations. It also aligns with studies highlighting the benefits of simulation and role-play in training processes, as these are active learning techniques that engage trainees in constructing their own knowledge and developing practical skills (Feinstein et al., 2002), while also improving confidence, knowledge, and readiness toward mental health interventions (Attoe et al., 2017; Richard et al., 2023).

Moreover, trainees not only acquired competencies to teach ER skills but also reported having integrated them into their daily lives, experiencing changes in how they approached personal and professional situations. This result aligns with previous research on socioemotional skills training programs (Jennings and Greenberg, 2009; Brackett et al., 2011), which shows that the effectiveness of training depends not only on transmitting strategies but also on whether trainers internalize them as part of their emotional repertoire. It also reinforces the ideas of Linehan (2015) and Bowden et al. (2021), who indicate that credibility and fidelity in teaching are strengthened when implementers teach from lived experience. The contribution of this study lies in documenting how this process evolves “from self to others”—that is, moving from self-regulation to co-regulation and teaching adolescents, representing a central transition in implementer preparation, a dimension rarely described in previous literature.

The relational dimension and the bonds formed among trainees and with trainers were recognized as fundamental throughout the training process, contributing significantly to the trainees’ learning. Building a climate of trust and openness facilitated trainees’ willingness to raise questions, express opinions about content, and share experiences without fear of judgment. In this regard, trust has been shown to influence learning environments and the development of professional competencies (Wisshak and Hochholdinger, 2018; Bonnie et al., 2020), while an inclusive climate enhances the acquisition of ER skills (Holmqvist Larsson et al., 2023; Evans et al., 2024). Therefore, the quality of the relationship with trainers appears to be a relevant factor not only for knowledge transfer but also for creating a safe space that fosters learning.

A key finding of this study is that investing time from the beginning to create a climate of trust between trainees and trainers allowed participants, in later phases, to take risks during piloting activities—such as role-plays—and to exchange constructive feedback. This suggests that preparation for implementing an intervention depends not only on theoretical content or supervised practice but also on relational conditions that facilitate the appropriation of competencies. Thus, these results align with the framework of competency drivers (Fixsen et al., 2005; Metz and Bartley, 2012), indicating that creating a safe environment is a critical element for enhancing both skill learning and implementation fidelity.

Several authors have noted that pilot studies and simulations aid in anticipating obstacles and enhance the feasibility and fidelity of interventions (Feeley et al., 2009; Day et al., 2014). The present findings complement this perspective by showing that trainees’ perceptions serve as a valuable source of feedback for program design. During the piloting phase, trainees identified potential challenges related to adolescent motivation, time management, and the possibility of emotional crises or sensitive disclosures. This forward-looking perspective suggests that systematically incorporating formative evaluation from trainees into program design not only reinforces existing evidence on implementation fidelity (Durlak and DuPre, 2008; Little et al., 2013) but also provides concrete inputs for preventive adjustments before the real intervention begins.

### 4.1 Limitations

This study was based on the perceptions of seven trainees who participated in a specific training process within a single implementation cohort, which limits the scope of the findings to this particular context and moment. While the small and context-specific sample constrains statistical generalizability, the study was designed to provide an in-depth qualitative exploration rather than broad representativeness. Accordingly, the results are not intended to be generalizable but rather transferable to similar contexts, depending on the characteristics and conditions of each implementation. Furthermore, the experiences collected correspond to a stage prior to the actual intervention with adolescents, and therefore do not include trainees’ perspectives after facing real school conditions. Future research could include follow-up assessments to examine how the perceptions and skills developed during training translate into actual intervention implementation, as well as to explore the impact of the training process on outcomes among adolescent participants.

### 4.2 Conclusion

Taken together, the findings identify four dimensions relevant to ER implementer training: critically assessing the training process; moving toward ownership of learning and becoming facilitators; valuing relationships as a foundation of the training experience; and anticipating challenges in the facilitator role. These dimensions highlight that implementer preparation extends beyond technical instruction and involves reflective, relational, and anticipatory processes that shape readiness for real-world delivery.

While these dimensions do not constitute definitive evidence of effectiveness, they offer empirically grounded insights for the design of future training programs. Based on these findings, several practical considerations may inform the design of implementer training programs. Investing time in building a safe and collaborative relational climate appears essential, as it enables trainees to experiment, express uncertainty, and engage in constructive feedback. When interventions involve socio-emotional skills, incorporating experiential components that enable implementers to internalize skills before teaching them may strengthen authenticity and confidence in delivery. Structured piloting and role-play activities that simulate realistic classroom challenges can facilitate the transition from personal learning to pedagogical practice. Finally, explicitly addressing anticipated contextual and crisis-related situations through clear protocols and shared reflection may enhance implementers’ preparedness and flexibility in school settings. Together, these elements may contribute to strengthening implementation fidelity and enhancing the sustainability of school-based socio-emotional interventions.

## Data Availability

The raw data supporting the conclusions of this article will be made available by the authors, without undue reservation.
