# Supplementary material for: Developing implementer competence: trainees’ experiences during a training program for an emotion regulation intervention in schools
Source: Front Psychol. 2026 Mar 24;17:1747220. doi: 10.3389/fpsyg.2026.1747220 (PMC13055588; doi:10.3389/fpsyg.2026.1747220)
Supplement: Supplementary file 1 [file Supplementary_file_1.docx]

Supplementary Material

# Appendix A. Semi-Structured Individual Interview Guide

| **Guiding Questions** | **Probing Questions** |
| --- | --- |
| How would you describe your experience in the Emotion Regulation Training?  Please describe the training process and your participation in it. What aspects stand out to you? | How much time did you invest in the training process?  How were the training sessions structured? (e.g., lecture-based, group discussion, experiential activities)  How would you characterize the group dynamics (e.g., trust, openness, interaction flow)?  Which aspects of the training methodology did you find most valuable?  Which activities would you identify as particularly relevant? Why?  Which components or resources provided during the process did you perceive as especially beneficial?  Which components or resources did you perceive as especially challenging?  Compared to previous training experiences, what differences do you identify?  How would you describe the support received from trainers and peers during the Emotion Regulation Training? In which areas could additional support have been beneficial  How did the role of the trainers influence the training process?  Which aspects of the training process could be improved for future interventionists?  How would you evaluate the closing phase of the training process in relation to its duration and content?  How would you describe your overall experience upon completing the training process? |
| What key learnings did you derive from participating in the Emotion Regulation Training? | Which learnings do you consider most significant?  To what extent do you consider these learnings to have been meaningful and substantial? Why?  How might these learnings contribute to your capacity to teach emotion regulation skills to adolescents in your role as an interventionist?  How would you describe your level of confidence in teaching emotion regulation skills to adolescents before and after the Emotion Regulation Training?  In what ways could the knowledge and skills acquired be applied to your personal and/or professional development? |
| Which personal characteristics or prior learning experiences facilitated your participation in the training process? | Which pre-existing skills or knowledge were particularly useful during the Emotion Regulation Training? Why?  To what extent did the Emotion Regulation Training expand your existing competencies or provide new skills?  Did you encounter specific challenges during the training process due to limited prior experience or competencies?  Which aspects of your participation were facilitated by your previous background or preparation? |
| *Thinking about implementing what you learned in the future:*  What challenges do you anticipate when implementing an emotion regulation intervention for adolescents? | Considering the developmental characteristics of adolescence, what potential challenges might arise during implementation?  What cultural or social factors could hinder the acceptance or effectiveness of this intervention in your community?  What strategies might be helpful in addressing potential implementation challenges?  Would you require additional support to implement the intervention? If so, what type?  How has the Emotion Regulation Training prepared you to address potential future challenges? |

# Appendix B. Semi-Structured Focus Group Interview Guide

| **Guiding Questions** | **Probing Questions** |
| --- | --- |
| How would you describe the second phase of the Emotion Regulation Training? (objectives, activities, who implemented it, who participated, how sessions were organized) | Please describe how a session in this phase was structured.  Among the activities carried out, which ones would you highlight? Why?  Which moments or resources provided during the process did you consider especially beneficial?  How would you define or characterize this second phase? |
| What roles did each participant have during these sessions? | What elements facilitated or hindered this process and the performance of each role?  What aspects were easy or difficult in performing each role? |
| Which elements of this phase were most helpful for you (both as interventionists and in reflecting on adolescents’ perspectives)?  Compared to the previous phase of the training process, what do you value most about this second phase?  What skills do you think you developed during this second phase?  How confident do you feel about implementing a future intervention? | What aspects of this phase could be improved in future implementations? |
| How would you describe your experience at the conclusion of this second phase?  Which competencies do you believe require further development to feel fully prepared for implementation?  What aspects of implementing the intervention do you anticipate will be most challenging? | Overall evaluation of the training process  What makes you feel prepared to implement the interventions?  Would additional resources or competencies be necessary to facilitate implementation? Please specify. |
